# Factors influencing the occurrence of ambulatory care sensitive conditions in the emergency department - a single-center cross-sectional study

**DOI:** 10.3389/fmed.2023.1256447

**Published:** 2023-11-09

**Authors:** Leo Benning, Jan Kleinekort, Michael Clemens Röttger, Nora Köhne, Julius Wehrle, Marco Blum, Hans-Jörg Busch, Felix Patricius Hans

**Affiliations:** ^1^University Emergency Department, University Medical Center Freiburg, Albert-Ludwigs-University Freiburg, Freiburg, Germany; ^2^Data Integration Center, University Medical Center Freiburg, Albert-Ludwigs-University Freiburg, Freiburg, Germany

**Keywords:** ambulatory care sensitive conditions, emergency medicine, health policy, health services accessibility, quality of healthcare, routinely collected health data

## Abstract

**Background and importance:**

The differentiation between patients who require urgent care and those who could receive adequate care through ambulatory services remains a challenge in managing patient volumes in emergency departments (ED). Different approaches were pursued to characterize patients that could safely divert to ambulatory care. However, this characterization remains challenging as the urgency upon presentation is assessed based on immediately available characteristics of the patients rather than on subsequent diagnoses. This work employs a core set of Ambulatory Care Sensitive Conditions (core-ACSCs) in an ED to describe conditions that do not require inpatient care if treated adequately in the ambulatory care sector. It subsequently analyzes the corresponding triage levels and admission status to determine whether core-ACSCs relevantly contribute to patient volumes in an ED.

**Settings and participants:**

Single center cross-sectional analysis of routine data of a tertiary ED in 2019.

**Outcome measures and analysis:**

The proportion of core-ACSCs among all presentations was assessed. Triage levels were binarily classified as “urgent” and “non-urgent,” and the distribution of core-ACSCs in both categories was studied. Additionally, the patients presenting with core-ACSCs requiring inpatient care were assessed based on adjusted residuals and logistic regression. The proportion being discharged home underwent further investigation.

**Main results:**

This study analyzed 43,382 cases of which 10.79% (*n* = 4,683) fell under the definition of core-ACSC categories. 65.2% of all core-ACSCs were urgent and received inpatient care in 62.8% of the urgent cases. 34.8% of the core-ACSCs were categorized as non-urgent, 92.4% of wich were discharged home. Age, triage level and sex significantly affected the odds of requiring hospital admission after presenting with core-ACSCs. The two core-ACSCs that mainly contributed to non-urgent cases discharged home after the presentation were “back pain” and “soft tissue disorders.”

**Discussion:**

Core-ACSCs contribute relevantly to overall ED patient volume but cannot be considered the primary drivers of crowding. However, once patients presented to the ED with what was later confirmed as a core-ACSC, they required urgent care in 65.2%. This finding highlights the importance of effective ambulatory care to avoid emergency presentations. Additionally, the core-ACSC categories “back pain” and “soft tissue disorders” were often found to be non-urgent and discharged home. Although further research is required, these core-ACSCs could be considered potentially avoidable ED presentations.

**Clinical trial registration:**

The study was registered in the German trials register (DRKS-ID: DRKS00029751) on 2022-07-22.

## Background/introduction

Like many European healthcare systems, the German system is coined by its relatively strict separation of inpatient and outpatient care (1). This setting poses challenges when the required care for a patient transgresses the area of responsibility of one sector to the other (i.e., either from inpatient to outpatient care or vice versa). In the specific context of the German healthcare system multiple initiatives have been introduced to bridge the interface between the two sectors (e.g., hospital-operated integrated outpatient care centers, off-hour urgent care practices and structured disease management programs), but barriers remain. These are partly related to historical reasons, due to which emergency and urgent care are primarily in the responsibility of the primary care providers, but also due to administrative reasons (1). For the latter, the presentation of any patient has to be assigned to either the inpatient, or the outpatient sector. By the very nature of emergency care, however, this circumstance often poses difficulties in guiding patients toward the appropriate care and is most challenging when an unscheduled presentation in an emergency department (ED) occurs.

EDs typically assign a triage level to all patients, assess their urgency, determine the allowed time intervals to be seen by ED staff, and estimate the resources needed. The most widely used triage systems in industrialized countries are the Canadian Triage and Acuity Scale (CTAS), the Emergency Severity Index (ESI) and the Machester Triage System (MTS) (2). Although these systems have conceptual differences, the common aim is to prioritize patient throughput in the ED by forming levels of urgency. In the presented 5-level triage systems, the highest priority is represented by level 1 (immediate life-saving measures required). The lowest priority, i.e., level 5, consequently indicates minimum risk and minimum resource intensity.

While an ED is typically staffed and equipped to attend to critically ill or injured patients, these resources are often utilized to treat non-urgent presentations. This phenomenon not only impairs the ability of an ED to meet its original responsibilities of providing emergency care but also leads to a high and excessive workload for healthcare workers. The effects are summarized under the concept of “Overcrowding” (3–5). In this light, the awareness of certain medical conditions that typically do not require inpatient or urgent care, but well-coordinated ambulatory or outpatient care, has continuously grown over the past years. This context has fostered the development of different concepts of ambulatory care sensitive conditions (ACSC) (6–10). These concepts have in common that they address diseases for which the likelihood of hospitalization can be reduced through timely and effective ambulatory care (11, 12). These health conditions are, however, severe enough that they could potentially require hospitalization and subsequent inpatient care if they are not appropriately managed in an outpatient setting (e.g., regular check-ups, screenings, and medication management).

A relevant proportion of patients presenting in the ED are supposed to fall under this category and should typically not require urgent or emergency care (6, 11, 13). It has remained unclear, however, whether these presentations are a consequence of the insufficient availability of or access to the required ambulatory care, the poor coordination of care at the intersection between the inpatient and outpatient sector, or whether they primarily represent acute and potentially critical exacerbations of otherwise sufficiently treated and controlled conditions. Thus, the analysis of triage levels and ACSCs poses the opportunity to identify potentially preventable presentations and, subsequently, hospitalizations. To explore this spectrum of conditions, this work applies the core list of ACSCs (core-ACSCs), as proposed by Sundmacher et al. (9), and compares its distribution across the triage levels and the requirement for inpatient care in the ED of a tertiary care facility in Germany.

## Aims

This study aims to identify the proportion of core-ACSCs in emergency patients of an academic tertiary hospital and analyze the urgency (triage level) of these conditions together with factors that may influence these attendances. Thereby, we aim to achieve insights into potentially preventable presentations to an ED.

## Methods

We obtained anonymous routine data of a tertiary German emergency department from the local data integration center after approval by the appropriate ethics committee (University of Freiburg, Ethics Committee number 21–1607) and the use and access committee (UAC). The study was registered in the German trials register (DRKS-ID: DRKS00029751) on 2022-07-22. By the anonymous nature of this data, the dataset does not fall under European data protection laws. Therefore, no individual consent was required, and no formal enrollment or recruitment was necessary. The study was conducted as a cross-sectional analysis of routinely collected demographic, triage and administrative data from adult patients who presented in the ED of a tertiary care university medical center in Germany in 2019.

The center is located in the southwest of Germany and is one of the largest university medical centers in Germany with approximately 90,000 patients receiving inpatient treatment and more than 900,000 patients receiving ambulatory care per year. Due to its location in proximity to the Swiss and French borders, it also provides specialty care for the border regions.

The categories in the dataset included age, sex, zip code, date of presentation, triage level, ICD-10-GM (German modification) diagnosis and inpatient status. Triage level was assigned according to the Emergency Severity Index (ESI) framework. While a comprehensive introduction to the ESI framework can be found elsewhere (14), it generally provides a 5-level triage system in which the highest priority is represented by level 1 (immediate life-saving measures required), whereas the lowest priority, i.e., level 5, consequently indicates minimum risk and minimum expected resource intensity. We employed the core list of ACSCs and the respective categorizations, as proposed by Sundmacher et al. (9). Wilcoxon rank-sum tests were used to assess discrete variables, whereas Chi-square tests were employed to determine distributions of categorical data. The distribution of core-ACSCs across different severity levels (i.e., “urgent,” ESI 1–3 and “non-urgent,” ESI 4–5, respectively) and admission status was displayed and assessed with Chi-square tests. Subsequently, a post-hoc analysis was performed using adjusted residuals. A logistic regression was calculated to assess the effect of patient characteristics available at the time of presentation (i.e., age, season, triage level, sex, country of residence, proximity to the emergency department by ZIP code) on the odds of the respective patients requiring admission to the hospital. A sensitivity analysis assessed the effect of the above-mentioned characteristics on the odds of a presentation yielding any core-ACSC.

Additionally, the proportion of “non-urgent” core-ACSCs not requiring admission/inpatient care was assessed for secondary analysis. Lastly, we investigated the ICD-10-GM codes within the latter subgroup of discharged patients. All calculations were rendered with Stata 17 (24).

## Results

There were 48,409 recorded patient contacts in the ED in 2019. 3,347 patients were under the age of majority (18 years) at admission and were excluded according to the study protocol. Another 1,667 datasets were excluded due to missing or implausible values for the emergency severity index (ESI), and another three due to a missing sex indicator. Further analysis of the dataset revealed two core-ACSC categories (diseases of the eye, sleep disorders), which occurred less than 10 times in the dataset and, therefore, were excluded. The final analysis of the incidence and urgency of the ASCS included the remaining 43,382 unique datasets (Table 1).

**Table 1:** Baseline characteristics of the 43,382 datasets from the tertiary emergency department included in the analysis.

Category (*N*, %)	ACSC (4,683, 10.79)	Non-ACSC (38,699, 89.21)	Hypothesis test
Age at time of admission in years (SD)	50,19 (20.35)	49,51 (21.17)	** (a)
Female gender (%)	49.37	45.00	*** (b)
Country of residence (%)	Germany: 98.38 Other: 1.62	Germany: 97.71 Other: 2.29	** (b) ** (b)
Triage level at the time of admission (%)	ESI 1: 1.79 ESI 2: 25.28 ESI 3: 38.14 ESI 4: 20.54 ESI 5: 14.24	ESI 1: 2.71 ESI 2: 17.33 ESI 3: 41.06 ESI 4: 27.33 ESI 5: 11.57	*** (b) *** (b) *** (b) *** (b) *** (b)

A Wilcoxon rank-sum test was used to assess discrete variables, whereas a Chi-square test was employed to determine distributions of categorical data. (a) Wilcoxon ranked-sum test; (b) Chi-squared test.

### General findings

4,683 (10.79%) of the patients obtained a diagnosis contained in the core-ACSC list by Sundmacher et al. (9), and 38,699 of the patients (89.21%) were in the non-ACSC group. Age at the time of admission was 50,19 (+/− 20,35) years in the ACSC group and 49,51 years (+/− 21,17) in the non-ACSC group, respectively (*p* = 0.0096). We also detected significant differences in the distribution of sex, country of residence and ESI levels at the time of presentation, as shown in Table 2.

**Table 2:** Core-ACSC modified after (1) with the percentage assigned to high triage levels (ESI 1–3).

No.	Core-ACSC	High triage level (i.e., ESI 1–3) (%)	Admitted / inpatient care required (*n*, adjusted residual ±)
1	Ischemic heart disease	66.32 (c)	63 (4.514) (d)
2	Heart failure	96.92 (c)	63 (8.773) (d)
3	Other diseases of the circulatory system	57.23 (c)	186 (5.143) (d)
4	Bronchitis and COPD	79.84 (c)	103 (8.423) (d)
5	Mental and behavioral disorders due to alcohol	70.07 (c)	281 (11.188) (d)
6	Back pain	21.36	188 (−14.751)
7	Hypertension	67.02 (c)	254 (9.597) (d)
8	Gastroenteritis	46.67	77 (0.813)
9	Intestinal infectious diseases	45.54	138 (0.712)
10	Influenza and pneumonia	70.09 (c)	150 (8.006) (d)
11	Ear, Nose, and Throat problems	18.36	38 (−7.487)
12	Depression	60.61 (c)	20 (1.979) (d)
13	Diabetes mellitus	75.81 (c)	47 (5.151) (d)
14	Gonarthrosis	9.09	4 (−4.636)
15	Soft tissue disorders	26.47	153 (−8.862)
16	Other avoidable mental and behavioral disorders	38.16	58 (−1.371)
17	Diseases of the eye §	-	-
18	Diseases of the urinary system	41.09	113 (−0.859)
19	Sleep disorders §	-	-
20	Diseases of the skin and subcutaneous tissue	35.24	74 (−2.495)
21	Malnutrition	73.08 (c)	19 (3.041) (d)
22	Dental diseases	8.57	12 (−8.482)

Proportions >50% of cases with high triage levels are indexed with (c). The numbers of admitted patients are given with adjusted residuals for comparison in brackets. The suffix (d) indicates significant skewness toward inpatient care of the core-ACSC. The enclosed ICD-10 are given in the Supplementary Table S1. ACSC with § occurred less than 10 times in the dataset and, therefore, were excluded. ± adjusted residuals: Z-scores greater than 1.96 or less than − 1.96 indicate a statistically significant difference from an equal distribution and exceed the 95%-CI of the expected distribution. Z-scores greater than 2.576 or less than − 2.576 exceed the 99%-CI, respectively.

### Distribution of ACSCs across triage levels

**Figure 1:**
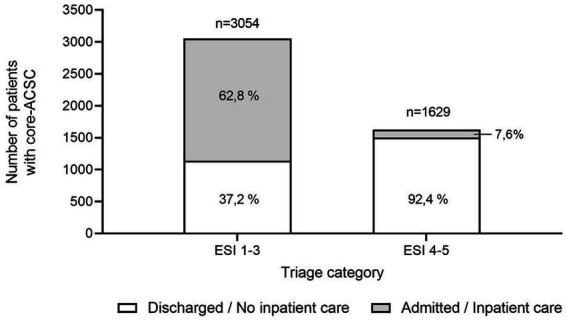
Triage levels and disposition status (discharge - no inpatient care required; admission or inpatient care required) of all core-ACSC (*n* = 4,683) in 2019. 65.2% (*n* = 3,054) of all core-ACSC were highly urgent (ESI1-3) and received inpatient care in 62.8% of the high triage level cases. 34.8% (*n* = 1,629) of the core-ACSC were attributed “non-urgent” (ESI 4–5) are subsequently discharged home in 92.4% of the cases.

The distribution of all core-ACSCs (*n* = 4,683) between “urgent” (ESI 1–3, *n* = 3′054) and “non-urgent” (ESI 4–5, *n* = 1,629) triage levels showed a relevant proportion of all core-ACSC categories to fall in high triage levels (65.2%). Of all patients with a high triage level at presentation, 62.8% were admitted or received inpatient care, whereas 37.2% were discharged home. Among the patients with a non-urgent triage level at the time of presentation, only 7.6% were admitted or received inpatient care, and 92.4% were discharged home, respectively (Figure 1).

11/20 core-ACSC categories were considered a high triage level in >50% of all presentations, indicating a higher urgency at the time of presentation (see (c) Table 2). In parallel, these ACSC categories resulted in significantly more admissions or inpatient treatment, as indicated by the adjusted residuals exceeding 1.96 (see (d) Table 2). No deviation from the pattern of a high proportion of high triage levels and a high proportion of admissions or inpatient treatments could be observed. The overall frequency of core-ACSC and the enclosed ICD-10 codes is provided in Supplementary Table S1.

### Influence of patient characteristics on the odds of presenting with ACSCs

Beyond the distribution among individual core-ACSCs, we analyzed patient and administrative data available at the time of presentation for their effect on the odds of a patient requiring hospital admission for the treatment of a core-ACSC. While older (OR 1.01, 95%-CI 1.007–1.012) and female patients (OR 1.14, 95%-CI 1.041–1.248) have significantly higher odds ratios for requiring a hospital admission after presenting with a core-ACSC, lower triage levels (ESI 4–5) (OR 0.46, 95%-CI 0.437–0.485) show significantly lower odds ratios for a subsequent admission (Table 3).

**Table 3:** Logistic regression of available patient and administrative characteristics at the time of presentation on the odds of requiring hospital admission from the ED after presenting with any core-ACSC.

Core-ACSC with resulting hospital admission from ED	Odds ratio	z	*p* > |z|	95%-CI
Patient and administrative characteristics				
Age	1.009796	8.53	<0.001	1.007537–1.01206
Season (a)	0.919878	−1.81	0.071	0.84016–1.00716
Triage level	0.4603967	−28.69	<0.001	0.4366357–0.4854507
Female	1.139787	2.83	0.005	1.040887–1.248085
Country of residency	1.181307	0.82	0.414	0.7917638–1.762503
Proximity to ED (b)	1.167186	1.86	0.063	0.9914136–1.374122
Baseline odds	0.204044	−7.39	0.000	0.1338608–0.3110244

(a) Season: Months of presentation were grouped as summer (1, i.e., April through September) and winter season (0, i.e., October through March).

(b) Proximity to ED: Truncated two-digit zip codes were used to assess regional proximity (i.e., presentation from within or from outside of the geographic region of the ED).

A sensitivity analysis assessed the effect of the above-mentioned patient and administrative characteristics on the odds of presenting with any core-ACSC. To the contrary of the analysis above, no significant effect of age (OR 0.99, 95%-CI 0.998–1.001) could be found, while patients with core-ACSCs presented significantly less often during the summer months (OR 0.64, 95%-CI 0.598–0.677) (Supplementary Table S2). Additionally, patients with any core-ACSC were significantly more often from the same geographic region of the ED than from other areas (OR 1.17, 95%-CI 1.056–1.311) (Supplementary Table S2).

### Non-urgent core-ACSCs with subsequent discharge

We further analyzed the patients who received a non-urgent triage level at presentation and were discharged home. Their distribution between the different core-ACSCs is displayed in Table 4. We identified that most non-urgent and discharged patients presented with back pain (32.93%) or soft tissue disorders (20.32%). Intestinal infectious diseases (5.38%), Ear, Nose, and Throat Problems (7.77%), diseases of the urinary tract system (6.44%), diseases of the skin and subcutaneous tissue (5.31%) and dental problems (7.9%) were also present to a relevant extent.

**Table 4:** Frequencies of core-ACSCs with non-urgent triage levels (ESI 4–5) and discharge home.

No.	Non-urgent core-ACSC with subsequent discharge	Frequency (*n*)	(%)
1	Ischaemic heart diseases	0	0
2	Heart failure	0	0
3	Other diseases of the circulatory system	37	2.46
4	Bronchitis and COPD	5	0.33
5	Mental and behavioral disorders due to alcohol	19	1.26
6	Back pain	496	32.93#
7	Hypertension	20	1.33
8	Gastroenteritis	30	1.99
9	Intestinal infectious diseases	81	5.38*
10	Influenza and pneumonia	10	0.66
11	Ear Nose and Throat problems	117	7.77*
12	Depression	7	0.46
13	Diabetes mellitus	3	0.20
14	Osteoarthritis of the knee/Gonarthrosis	29	1.93
15	Soft tissue disorders	306	20.32#
16	Other avoidable mental/behavioral disorders	49	3.25
17	Diseases of the eye§	-	-
18	Diseases of the urinary system	97	6.44*
19	Sleep disorders§	-	-
20	Diseases of the skin and subcutaneous tissue	80	5.31*
21	Malnutrition	1	0.07
22	Dental diseases	119	7.90*
	**Total**	**1,506**	**100**

The two core-ACSCs that contribute >10% to this subpopulation are highlighted with # (see Table 5), and core-ACSCs >5% with an *. The enclosed ICD-10 are given in the Supplementary Table S1. ACSCs with § occurred less than 10 times in the full dataset and were, therefore, excluded.

**Table 5:** ICD-10-GM, within the two core-ACSC groups that contribute >50% to non-urgent triage levels and are discharged home from the ED without needing inpatient care.

No.	Non-urgent core-ACSCs with subsequent discharge	ICD-10	Frequency (*n*)	(%)
6	Back pain	M47 (Spondylosis)	8	1.61
		M53 (Other dorsopathies)	15	3.02
		M54 (Dorsalgia)	473	95.36
15	Soft tissue disorders	G56 (Mononeuropathies of the upper limb)	5	1.63
		M67 (Other disorders of synovium and tendon)	1	0.33
		M75 (Shoulder lesions)	22	7.19
		M76 (Enthesopathies of the lower limb, excluding foot)	19	6.21
		M77 (Other enthesopathies)	19	6.21
		M79 (Other soft tissue disorders, not elsewhere classified)	240	78.43

### Potentially preventable core-ACSC presentations

A third level of the analysis examined the ICD-10-GM codes that were grouped in the core-ACSCs that contributed to the subpopulation with non-urgent triage level at the time of presentation and discharge home (i.e., back pain and soft tissue disorders). Among those, specifically, the dorsalgia (M54) was diagnosed most often and accounted for 95.36% (473/496) of all patients from the core-ACSCs with back pain and 31.4% (473/1,506) of all patients with a non-urgent triage level at the time of presentation and subsequent discharge home. While the category of soft tissue disorders was distributed more widely, 78.43% (240/306) received the diagnosis of other soft tissue disorders (M79). At the same time, enthesopathies (M76, M77) were diagnosed in 12.42% (38/306) and shoulder lesions (M75) in 7.19% (22/306) of the cases, respectively.

## Discussion

This analysis is based on cross-sectional routine data from a tertiary care level ED in Germany. It assesses the relationship between triage levels assigned at the time of presentation and resulting admission or discharge diagnoses in a catalog of ACSCs established for the German healthcare system. By the nature of the study design, it could not establish causal relationships but provided important exploratory insights into the role of core-ACSCs in emergency care.

Patients presenting with core-ACSCs and non-ACSC diagnoses differ significantly regarding their baseline characteristics of age, sex, country of residence and triage level. However, the magnitudes of these differences do not suggest a clinically relevant difference between the patient populations (Table 1). Furthermore, and as hypothesized, core-ACSCs account for a relevant proportion of ED visits (10.79%). This finding aligns well with prior findings from other health systems (15). However, 65.2% (3,054/4,683) of these did actually require urgent care, as indicated by high triage levels (ESI 1–3; “urgent”) at the time of presentation (Figure 1). Therefore, and contrary to prior assumptions (16), they could not be plausibly considered a driver for unjustified ED volumes and crowding. Especially cardiovascular, respiratory and metabolic core-ACSCs often led to urgent presentations in the ED and required subsequent admission or inpatient care (Table 1). Thus, it is doubtful if these conditions - once they are presented in the ED - could be managed adequately in the ambulatory care sector. This finding underscores the importance of timely and effective ambulatory care to avoid the presentation of these patients as emergencies and to facilitate a coordinated admission to inpatient care once it is required.

An additional regression analysis found that higher age contributes to significantly higher odds of requiring admission (Table 3), which aligns with prior research in this field (17). This finding could be due to the increased complexity of older patients in the context of multimorbidity, specific care needs, or a decreased physical reserve with an overall higher risk for a poor outcome from otherwise manageable conditions (18). Yet, this analysis could not account for the latter factors due to the limited availability of structured data.

Similarly, female patients have higher odds of requiring hospitalization after presenting with a core-ACSC (Table 3). While similar findings have also been reported before (19), our analysis again warrants a careful interpretation due to the lack of sufficient data on reported symptoms and comorbidities. A sensitivity analysis provided further insights and suggests that patients with core-ACSCs present less likely during the summer months and are more often from the same geographic region of the ED (Supplementary Table S2). These findings suggest that during the winter months (i.e., when ambulatory care services face higher workloads due to seasonal peaks of, for example, flu-like illnesses) core-ACSCs might not be managed effectively in the ambulatory care sector.

Interestingly, however, we also identified a group of presentations with non-urgent triage levels (ESI4-5) that often leads to a subsequent discharge back home and into the ambulatory care sector, respectively. This group consisted primarily of musculoskeletal conditions, namely dorsalgia and a number of minor soft tissue disorders (i.e., non-specific soft tissue disorders, enthesopathies and shoulder lesions). Although the available data do not reveal whether adequate care for these patients required specific resources that are only readily available in an ED (e.g., radiologic diagnostics or intravenous analgesia), the results (i.e., “non-urgency” and timely discharge) suggest that certain conditions can be considered potentially avoidable presentations to the ED.

Significant limitations to this analysis stem from the use of routine data, which - by nature - were not recorded for scientific purposes. Firstly, fluctuating data quality and low internal validity are typically flaws of this type of real-world data, while broader generalizability is among the advantages (20). Secondly, important meta-information on the selected cases (i.e., pain scores, drug therapy, concurrent diagnosis, comorbidities or socioeconomic background) was unavailable in the routine data. Therefore, no conclusions regarding the resource consumption in the ED or the fraction of equity-deserving groups in the dataset can be made so far. Whether patients with ACSCs use different resources in the ED than they would in the ambulatory care setting, thus, remains subject to future research.

Thirdly, the data of minors were excluded according to the study protocol as only children with severe trauma are regularly treated in the respective ED for adult patients. No conclusions on the distribution of ACSCs in this age group can be made, respectively.

Fourthly, data collected before the COVID-19 pandemic will likely only partially represent current dynamics in patient presentation patterns and volumes (21). A multicenter cross-sectional data analysis from post-pandemic circumstances would greatly benefit both aspects.

Lastly, and although this study could identify potentially preventable presentations from some core-ACSCs, it has become clear that there is a need for a more comprehensive set of indicators to fully assess the actual reason for the presentation (i.e., presenting complaints in addition to triage levels), resource consumption throughout the treatment in the ED (e.g., need for extended diagnostics, consultations from specialists) and cost data related to these presentations. The latter is crucial as the administrative data alone do not allow a precise differentiation between admission to a ward (i.e., admission in the narrow sense) and an extended stay in the ED (i.e., admission in the broader sense, or - as described above - inpatient treatment).

It should also be considered that the ACSC framework is limited to specific diagnoses and does neither factor in patient demographics, nor any context factors of the presentation, which limits the applicability of ACSCs as an instrument to identify patients for, e.g., patient discharge from the emergency department (15). Comprehensive prevention quality indicators (PQI) have been developed to identify the quality of ambulatory care in advance and after hospital visits using the ACSC framework (22). For Emergency Departments, the ED-PQI framework differentiates between acute and chronic ACSCs and other preventable conditions (23).

Yet, further research is needed to integrate these frameworks into the context of German EDs. Additionally, resource consumption and cost analysis would help to increase our findings’ generalizability.

## Conclusion

This work connects the framework of ACSC with triage levels at the time of presentation in a tertiary care ED for the first time. Although ACSCs contribute a relevant proportion of ED presentations, they can not be considered the primary driver of patient volume. Hence, they do not contribute excessively to ED crowding, as initially hypothesized. Due to the high urgency of some ACSCs at the time of presentation, the respective patients unquestionably do require inpatient care. This finding underscores the importance of early and adequate ambulatory care for managing ACSCs. Nonetheless, certain ACSCs are often discharged home after an initial assessment. While the ACSC concept suggests its applicability to identify these attendances, it is not comprehensive enough to clearly distinguish between urgent and non-urgent ED presentations. In combination with other approaches, as for example meaningful quality indicators, a better understanding of high patient volumes and resulting resource consumption could be achieved. Yet, the identification and implementation of such integrated approaches requires further research to identify possibly preventable ED presentations.

## Data availability statement

The raw data supporting the conclusions of this article will be made available by the authors, without undue reservation.

## Ethics statement

The studies involving humans were approved by University of Freiburg, Ethics Committee, Ethik-Kommission der Albert-Ludwigs-Universität Freiburg Engelberger Straße 21 79106 Freiburg-Germany. The studies were conducted in accordance with the local legislation and institutional requirements. The ethics committee/institutional review board waived the requirement of written informed consent for participation from the participants or the participants’ legal guardians/next of kin because Only anonymized data was obtained by the data integration center after ethics approval and use and access (UAC) approval.

## Author contributions

LB: Conceptualization, Data curation, Investigation, Supervision, Validation, Writing – original draft, Writing – review & editing, Formal analysis, Methodology, Software. JK: Data curation, Software, Writing – review & editing. MR: Data curation, Software, Writing – review & editing. NK: Conceptualization, Investigation, Writing – review & editing, Methodology. JW: Data curation, Investigation, Software, Writing – review & editing. MB: Data curation, Software, Writing – review & editing. H-JB: Writing – review & editing. FH: Conceptualization, Data curation, Funding acquisition, Investigation, Project administration, Resources, Supervision, Validation, Writing – original draft, Writing – review & editing.
