# Supplementary material for: Factors influencing the occurrence of ambulatory care sensitive conditions in the emergency department - a single-center cross-sectional study
Source: Front Med (Lausanne). 2023 Nov 9;10:1256447. doi: 10.3389/fmed.2023.1256447 (PMC10665907; doi:10.3389/fmed.2023.1256447)
Supplement: Supplementary file 1 [file Table_1.docx]

Supplementary Table 1

Supplementary Table 1: 22 Core-ambulatory care-sensitive conditions [[9]](https://www.zotero.org/google-docs/?0fkWoR) with their occurrence in the analyzed dataset.

| **No.** | **Core-ACSC** | **Enclosed ICD-10** | **Count (%)** |
| --- | --- | --- | --- |
| 1 | Ischaemic heart diseases | I20, I25.0, I25.1, I25.5, I25.6, I25.8, I25.9 | 95 (2,03) |
| 2 | Heart failure | I50 | 65 (1,39) |
| 3 | Other diseases of the circulatory system | I05, I06, I08.0, I49.8, I49.9, I67.2, I67.4, I70, I73, I78, I80.0, I80.80, I83, I86, I87, I95, R00.0, R00.2, R47.0 | 325 (6,94) |
| 4 | Bronchitis & COPD | J20, J21, J40-J44, J47 | 129 (2,75) |
| 5 | Mental and behavioral disorders due to use of alcohol or opioids | F10, F11 | 401 (8,56) |
| 6 | Back pain | M42, M47, M53, M54 | 880 (18,79) |
| 7 | Hypertension | I10-I15 | 379 (8,09) |
| 8 | Gastroenteritis and other diseases of intestines | K52.2, K52.8, K52.9, K57, K58, K59.0 | 165 (3,52) |
| 9 | Intestinal infectious diseases | A01, A02, A04, A05, A07-A09 | 303 (6,47) |
| 10 | Influenza and pneumonia | J10, J11, J13, J14, J15.3, J15.4, J15.7, J15.8, J15.9, J16.8, J18.0, J18.1, J18.8, J18.9 | 214 (4,57) |
| 11 | Ear nose throat infections | H66, J01-J03, J06, J31, J32, J35 | 207 (4,42) |
| 12 | Depressive disorders | F32, F33 | 33 (0,70) |
| 13 | Diabetes mellitus | E10.2-E10.6, E10.8, E10.9, E11, E13.6, E13.7, E13.9, E14, E16.2 | 62 (1,32) |
| 14 | Gonarthrosis | M17.0, M17.1, M17.4, M17.5, M17.9 | 44 (0,94) |
| 15 | Soft tissue disorders | G56.0, M67.4, M71.3, M75-M77, M79 | 578 (12,34) |
| 16 | Other avoidable mental and behavioural disorders | F40, F41, F43, F45, F50.0, F50.2, F60 | 152 (3,25) |
| 17 | Diseases of the eye | H25, H40 | 0 |
| 18 | Diseases of urinary system | N30, N34, N39.0 | 275 (5,87) |
| 19 | Sleep disorders | G47 | 0 |
| 20 | Diseases of the skin and subcutaneous tissue | A46, L01, L02, L04, L08.0, L08.8, L08.9, L60.0, L72.1, L98.0 | 210 (4,48) |
| 21 | Malnutrition & nutritional deficiencies | D50, D51-D52, D53.1, D56E40-64, R63.6 | 26 (0,56) |
| 22 | Dental diseases | K02, K04-K06, K08, K12, K13 | 140 (2,99) |
| Sum | | | 4´683 (100) |
