# Supplementary material for: Factors influencing the occurrence of ambulatory care sensitive conditions in the emergency department - a single-center cross-sectional study
Source: Front Med (Lausanne). 2023 Nov 9;10:1256447. doi: 10.3389/fmed.2023.1256447 (PMC10665907; doi:10.3389/fmed.2023.1256447)
Supplement: Supplementary file 2 [file Table_2.docx]

Supplementary Table 2

Supplementary Table 2: Logistic regression of available patient and administrative characteristics at time of presentation on the odds of presenting with any core-ACSC.

| **Presentation with any core-ACSC** | **Odds Ratio** | **z** | **P > \|z\|** | **95%-CI** |
| --- | --- | --- | --- | --- |
| Patient and administrative characteristics |  |  |  |  |
| Age | 0.9998731 | -0.16 | 0.870 | 0.9983503-1.001398 |
| Season (a) | 0.636407 | -14.37 | <0.001 | 0.5983618-0.6768712 |
| Triage level | 0.9312465 | -4.21 | <0.001 | 0.9008908-0.9626251 |
| Female | 1.190867 | 5.62 | <0.001 | 1.120472-1.265685 |
| Country of residency | 1.163656 | 1.16 | 0.246 | 0.9005539-1.503624 |
| Proximity to ED (b) | 1.17642 | 2.95 | 0.003 | 1.055908-1.310685 |
| Baseline odds | 0.1301736 | -14.31 | <0.001 | 0.0984649-0.1720935 |

1. Season: Months of presentation were grouped as summer (1, i.e., April through September) and winter season (0, i.e., October through March).
2. Proximity to ED: Truncated two-digit zip codes were used to assess regional proximity (i.e., presentation from within or from outside of the geographic region of the ED)
